# Transfer of Ochratoxin A into Tea and Coffee Beverages 

**DOI:** 10.3390/toxins6123438

**Published:** 2014-12-17

**Authors:** Frantisek Malir, Vladimir Ostry, Annie Pfohl-Leszkowicz, Jakub Toman, Ingrid Bazin, Tomas Roubal

**Affiliations:** 1 Department of Biology, Faculty of Science, University of Hradec Kralove, 50003 Hradec Kralove, Czech Republic; E-Mail: frantisek.malir@uhk.cz; 2 National Reference Center for Microfungi and Mycotoxins in Food Chains, Center of Health, Nutrition and Food in Brno, National Institute of Public Health in Prague, 61242 Brno, Czech Republic; E-Mail: ostry@chpr.szu.cz; 3 Department Bioprocess & Microbial Systems, Laboratory Chemical Engineering, INP/ENSA Toulouse, University of Toulouse, UMR 5503 CNRS/INPT/UPS, 31320 Auzeville-Tolosane, France; E-Mail: leszkowicz@ensat.fr; 4 Ecole des mines d’Ales, 6 av de Clavieres, 30100 Ales Cedex, France; E-Mail: Ingrid.Bazin@mines-ales.fr; 5 National Reference Laboratory for Biomarkers of Mycotoxins and Mycotoxins in Food, Institute of Public Health in Usti nad Labem, Regional Branch Hradec Kralove, 50002 Hradec Kralove, Czech Republic; E-Mail: tomas.roubal@zuusti.cz

**Keywords:** ochratoxin A, OTA, black tea, fruit tea, coffee, OTA transfer into beverages

## Abstract

Ochratoxin A (OTA) is nephrotoxic, hepatotoxic, immunotoxic, neurotoxic, reprotoxic, teratogenic, and carcinogenic (group 2B), being characterized by species and sex differences in sensitivity. Despite the fact that OTA is in some aspects a controversial topic, OTA is the most powerful renal carcinogen. The aim of this study was to make a small survey concerning OTA content in black tea, fruit tea, and ground roasted coffee, and to assess OTA transfer into beverages. OTA content was measured using a validated and accredited HPLC-FLD method with a limit of quantification (LOQ) of 0.35 ng/g. The OTA amount ranged from LOQ up to 250 ng/g in black tea and up to 104 ng/g in fruit tea. Black tea and fruit tea, naturally contaminated, were used to prepare tea infusions. The transfer from black tea to the infusion was 34.8% ± 1.3% and from fruit tea 4.1% ± 0.2%. Ground roasted coffee naturally contaminated at 0.92 ng/g was used to prepare seven kinds of coffee beverages. Depending on the type of process used, OTA transfer into coffee ranged from 22.3% to 66.1%. OTA intakes from fruit and black tea or coffee represent a non-negligible human source.

## 1. Introduction

Drinking either tea or coffee is a long tradition and part of many lifestyles. Coffee and tea drinks are popular and their consumption is growing all over the world. The proven health benefits of coffee amply justify considering this drink as a functional food [1]. Recently it has been mentioned that coffee is the most frequently consumed functional food worldwide [2]. Tea and coffee drinks are recognized for their stimulatory effects linked to caffeine and theine as well as to a range of other beneficial substances, such as phenolic compounds—flavonoids (e.g., catechins and anthocyanins) have antioxidant properties [3,4,5] or other bioactive components [3,6,7]. However, these infusions may also contain undesirable compounds including toxigenic moulds and their secondary metabolites—including mycotoxins such as ochratoxin A (OTA).

OTA is a widespread mycotoxin with several toxic effects [8,9,10]. OTA is one of the most deleterious mycotoxins [11,12]. OTA is produced worldwide in foodstuffs (including cereals, cocoa, soya, beans, nuts, spices such as red pepper, liquorice, grapes, beer, wine, green coffee, and roasted coffee) either by *Aspergillus* Section *Circumdati* (*A. ochraceus*, *A. westerdijkiae*, *A. steynii*) or *Aspergillus* Section *Nigri* (*A. carbonarius*, *A. foetidus*, *A. lacticoffeatus*, *A. niger*, *A. sclerotioniger*, *A. tubingensis*), mostly in subtropical and tropical areas [13,14,15,16,17,18,19,20,21], or *Penicillia*, especially *Penicillium verrucosum* and *P. nordicum* [19,22]. Herbal teas, if improperly stored, could be a substrate for mold growth, which can represent a non-negligible part of the human OTA dietary intake [23]. 

Various kinds of fungi are observed in teas [24,25,26,27,28,29,30,31,32,33,34]. *Aspergillus niger* has been associated with black tea [27]. *Aspergillus* Section *Nigri*, including *Aspergillus niger*, *A. acidus*, *A. awamori*, *A. tubingensis*, and *A. carbonarius*, were found in herbal teas available on the Swiss market. It was confirmed that 7% of *A. niger* isolates from herbal teas are able to produce OTA [33].

OTA has also been detected in both green and roasted coffee beans in different countries [17,35,36,37,38,39,40,41,42,43,44,45,46,47,48]. OTA contamination can be minimized by applying good agricultural practice and post-harvest handling, consisting of appropriate techniques of drying, grading, transportation, and storage of raw coffee [49,50]. The industrial processes (roasting) can significantly reduce the OTA amount but this decreases strongly depends on the initial OTA contamination and on the botanical variety of coffee beans [50,51].

Climate change’s effect on the mycotoxin content of coffee [49] and agricultural practices (organic *versus* non-organic agriculture) [52] require urgent consideration. As OTA is not significantly reduced by roasting, coffee drinks could be an important OTA source in the human diet [17,37,53,54,55]. A similar assumption can be made in respect to tea beverages.

Altogether, tea and coffee can be contaminated by toxigenic fungi [13,15,20,21,30,42,56,57,58,59,60,61] decreasing the quality of teas and coffee [30,56,57,58] and representing a risk of contamination by some mycotoxins, e.g., OTA [41]. 

In the Czech Republic, *Aspergillus flavus* was detected in black tea [28,29,30]. In another study conducted in the Czech Republic, the microfungal contamination of various types of teas including black, green, and herbal teas was investigated. In this latter study, 81 species of microfungi were detected [30]. *Aspergillus niger* agg. was found to be the most frequent in addition to *Aspergillus ochraceus* [30]; all of them are potential producers of OTA. 

Findings of *Aspergillus niger* and *A. ochraceus* in teas and coffees in the Czech Republic led to the decision to monitor OTA occurrence in raw tea and coffee beans, and to study the possible OTA transfer into tea and coffee infusions. As already observed for caffeine, the OTA content will be dependent on the method of preparation of the hot drinks [54,62]. Therefore, the aim of this study was to analyze the occurence of OTA in some samples of fruit teas, black teas, and ground roasted coffee, and to assess the OTA transfer into corresponding beverages according to their preparation. The data obtained will serve to assess the real OTA intake via tea and coffee drinks in the Czech population. 

## 2. Results and Discussion

### 2.1. OTA Content in Raw Black Teas and Fruit Teas

Twelve samples of black tea and 12 samples of fruit tea have been analyzed for their OTA content. Four samples of black tea out of 12 (25%) contained OTA above 0.35 ng/g. The concentrations in the contaminated samples were 1.85, 56.7, 86.6, and 250 ng/g, respectively. The arithmetic mean was 33.1 ng OTA/g of tea and the median value was 0.18 ng/g, considering by convention a concentration of OTA below 0.35 ng/g as half of the limit of quantification (LOQ) = 0.175 ng/g. 

Four samples of fruit tea out of 12 (25%) contained OTA more than 0.35 ng/g. The concentrations in the contaminated samples were 1.1, 1.4, 1.8, and 104 ng/g respectively. The arithmetic mean and the median value are 9.1 ng OTA/g and 0.18 ng/g, respectively. 

### 2.2. OTA Transfer into Tea Infusions

Only naturally contaminated samples served to make the infusions. No confirmation using artificially contaminated samples has been performed. Infusions were made from six portions of naturally contaminated black and fruit teas. Data obtained after the infusion from the most naturally contaminated black tea (250 ng/g) and after the infusion of the most naturally contaminated fruit tea sample (104 ng/g) are summarized in Table 1.

**Table 1:** OTA transfer from raw black and fruit tea to tea brews (%).

Summary statistics	Black tea	Fruit tea
arithmetic mean	34.8	4.1
median	34.8	4.1
90% percentile	36.2	4.3
SD	1.3	0.2

SD = standard deviation.

This means that about 152.3 ng OTA/250 mL can be found in a cup of black tea made from the most contaminated sample. A similar percentage of OTA transfer (data not shown) was obtained from the least contaminated black tea (e.g., at a concentration of 1.85 ng/g). The OTA transfer into a fruit tea infusion from the most contaminated sample (104 ng/g) was much lower (7.5 ng/250 mL). One explanation of lower OTA transfer into fruit tea infusions can be due to the lowering of the pH and/or to the presence of some compounds interfering with the transfer (see below). 

### 2.3. OTA Content in Ground Roasted Coffee

OTA has been analysed in 48 samples of ground roasted coffee. Out of 48 samples of coffee, 31 contained OTA above 0.35 ng/g. The highest concentration was 1.37 ng/g. The arithmetic mean was 0.51 ng OTA/g and the median value was 0.48 ng/g (considering by convention a concentration of OTA below 0.35 ng/g as half of the limit of quantification (LOQ) = 0.175 ng/g). Samples of coffee contaminated by OTA were used to prepare a sufficient amount of a homogeneous sample [63] of roasted ground coffee contaminated in the concentration of 0.92 ng/g. This sample was subsequently used for the preparation of the coffee drinks. 

### 2.4. OTA Transfer into Coffee Beverages

For preparing coffee drinks several techniques were used. All of them basically involved boiling ground roasted coffee in water or, alternatively, pouring, dripping, or spraying hot water through ground roasted coffee and then filtering [64].

The naturally contaminated ground roasted coffee sample (0.92 ng/g) was used to prepare the coffee beverages (six times for each process) according to seven different kinds of processes (see Section 2.4 and Table 2 and Table 3).

OTA transfer to the coffee drinks called “False Turkish coffee” and “Turkish coffee” is shown in Table 2.

**Table 2:** OTA transfer from ground roasted coffee to coffee beverage (%).

Summary statistics	False Turkish coffee	Turkish coffee
arithmetic mean	66.1	51.7
median	66.2	51.8
90% percentile	68.0	53.8
SD	1.8	2.1

SD = standard deviation.

The first type of coffee drink, “*false Turkish coffee*,” is comparable to “French press coffee,” but unlike the French press, in the Czech false Turkish coffee the ground coffee stayed at the bottom of the cup. After 10 min the brew was filtered and then OTA was extracted and purified on an imunoaffinity column. False Turkish coffee is most common and most frequently drunk in the Czech Republic. The amount of OTA transferred into false Turkish coffee was higher, due to the fact that the coffee cake staying at the bottom of a cup lengthened the contact with water. The amount of water and the time of contact passing through the cake play an important role in the amount of OTA in the coffee beverage.

Percentages of OTA transferred into the drinks made with an electric coffee machine are presented in Table 3.

**Table 3:** OTA transfer according to preparation of different kinds of coffee drinks (%).

Summary statistics	Lungo	Americano	Espresso	Doppio	Ristretto
arithmetic mean	54.5	50.8	32.2	30.2	22.3
median	54.9	51.1	32.9	29.8	22.7
90% percentile	56.9	53.0	35.8	32.8	25.9
SD	2.4	2.3	3.7	2.2	3.6

SD = standard deviation.

A higher quantity of OTA was transferred into Lungo and Americano compared to Espresso, Doppio, and Ristretto. The relation between the amount of OTA transferred and the volume of boiling water used to prepare the coffee drinks is presented in Table 4. 

**Table 4:** OTA transfer to different coffee drinks according to the volume of water.

Coffee drinks	Volume of boiling water (mL)	Transfer OTA from ground coffee to coffee drink (%)	Amount of OTA (ng/cup of coffee)
False Turkish coffee	100	66.1	4.26
Lungo	80	54.5	3.51
Turkish coffee	100	51.7	3.33
Americano	100	50.8	3.27
Espresso	30	32.2	2.07
Doppio	50	30.2	3.89
Ristretto	20	22.3	1.44

Studies on OTA transfer to coffee drinks have already been conducted, although their design was somewhat different [17,55]. More than 80% of OTA was transferred from artificially contaminated ground coffee to the infusions [55]. The percentage of OTA transferred from naturally contaminated roasted coffee ranged from 20% to 140%, depending on the type of coffee and the process of brewing [17]. Smaller volumes of water were used and the time of contact with the coffee was shorter in our study compared to the two others. The amount of OTA in coffee drinks increases when the time of contact is longer. This can partially explain a lower OTA transfer.

The amount (or volume) of water used is one of the most important factors in the transfer of OTA, but it is not the only one. In the case of Turkish coffee, even though the same amount of water was used as for false Turkish coffee, the amount of OTA was lower. This can be explained by the fact that Turkish coffee was made with cold water and brought slowly to a boil three times, resulting in the isomerization of OTA, which is no longer detected [17]. Interestingly, these OTA derivatives have been detected on the HPLC, but in the absence of a standard it was not possible to quantify it (data not shown). It should be remembered, however, that the disappearance (conversion) of OTA does not necessarily mean an absence or decrease in toxicity risk, as the decomposition products may be as dangerous as the parent OTA molecule itself. The amount of OTA was also dependent on the amount of ground coffee used. Doppio coffee, for which the coffee drink was prepared from 14 g of ground roasted coffee (instead of 7 g, as for the other beverages) and the total volume of water was 50 mL, contained more OTA than the other coffees. In Doppio, the coffee/water ratio corresponded to 7 g/25 mL, which is just in between Ristretto (7 g/20 mL) and Espresso (7 g/30 mL). Indeed, in 25 mL of Doppio the amount of OTA was 1.94 ng. This confirmed a direct link between OTA amount and the quantity of water used. Comparison between Ristretto and Espresso showed that a lower amount of OTA was found in Ristretto (20 mL/1.44 ng) compared to Espresso (30 mL/2.07 ng). The transfer of OTA into coffee can be influenced by the pH (see below). 

### 2.5. Measurement of the pH of the Beverages and Impact on OTA Transfer

One explaination of the low OTA transfer into fruit tea infusions compared to black tea infusions and coffee could be due to the pH of the beverages. OTA is a weak acid having two pka (4 and 7), and thus OTA is more soluble in aqueous phase when the pH is above 7. During infusion several compounds such as chlorogenic acid and aliphatic acids in coffee, organic acid in fruit tea, and organic acid as citric acid in black teas, are simultaneously extracted, lowering the pH. To test this hypothesis, the pH of the initial drinking water and of the beverages was measured in the same conditions. The pH was measured three times in normal drinking water, at temperatures between 25.2 and 25.5 °C, before boiling. The pH values ranged from 7.37 to 7.67, with an arithmetic mean pH of 7.54. Subsequently, the pH of normal drinking water following boiling was recorded three times, after cooling to temperatures between 25.1 and 28.8 °C. The pH values ranged from 8.25 to 8.30, with an arithmetic mean pH of 8.28. It is noteworthy that boiling action increased the pH of the water. The pH in black, fruit teas, and coffee beverages after cooling was recorded in Table 5.

**Table 5:** pH in samples of black tea, fruit tea, and coffee beverages.

Sample type	pH (mean ± SD)	temperature (°C)
Black tea beverages	6.68 ± 0.01	27.0
Fruit tea beverages	3.43 ± 0.01	28.8
False Turkish coffee	5.91 ± 0.01	28.9
Turkish coffee	5.34 ± 0.01	25.7
Lungo	5.13 ± 0.01	27.7
Americano	5.33 ± 0.01	27.3
Espresso	5.10 ± 0.01	24.0
Doppio	4.99 ± 0.01	26.2
Ristretto	4.98 ± 0.01	25.5

Characteristics as mean and SD were identified on the basis of measurements three times.

In both kinds of tea (black tea and fruit tea), the pHs are acidic. The acidity was more pronounced in the fruit tea (arithmetic mean 3.43 *versus* 6.68). The variation of the pH can explain the fluctuation of OTA transfer. When the pH decreases, the amount of OTA extracted in water is less likely to be high. The compounds responsible for the acidification are organic acids including citric acid. In the same way, the pHs of the coffee drinks were recorded. As for teas, coffee beverages are more acidic than the initial water. This is due to the extraction of chlorogenic acid [65,66]. 

### 2.6. OTA Intake Estimation via Hot Drinks

For an adult (60 kg bw) drinking one cup (250 mL) of the most contaminated black tea, the intake will be 2.5 ng OTA/kg bw, which corresponds to half of the safe dose. One cup of the most contaminated fruit tea will bring 0.125 ng OTA/kg bw, which can be considered as negligible.

In the case of coffee contaminated by 0.92 ng OTA/g, ingestion of one cup of Doppio led to an OTA intake of 0.065 ng/kg bw.

This latter data is in line with the data recently obtained in the research project No. NT 12051-3/2011 entitled “Ochratoxin A—health risk assessment for selected population groups in the Czech Republic,” which studied the dietary exposure and health risk characterization of OTA for 10 population groups of both sexes aged 4–90 years during 2011–2013. It has been found that coffee is the main source of OTA dietary intake in a group of Czech women aged 18–59, in comparison with other groups of the Czech population. Based on these results, OTA coffee intake for an “average consumer” in the Czech Republic was 0.06 ng/kg bw/day [67]. 

Nevertheless, it should be kept in mind that in general people drink more than one cup of tea or coffee per day. As can be observed in Table 4, consumption of black tea, and in some cases fruit tea, represent a non-negligible proportion of the tolerable daily intake.

Table 6 shows the mean daily intake and contribution of each hot drink analyzed to OTA’s tolerable daily intake (TDI) of 14 ng/kg bw/day and the virtually safe dose (VSD) of 5 ng/kg bw/day, as calculated by IARC and Health Canada [68]. For calculation purposes, daily ingestion of five cups of coffee or two cups of black tea or fruit infusion was taken into account.

**Table 6:** OTA daily intake contribution of each drink.

Type of drink	OTA intake (ng/kg bw/day)		Contribution to tolerable daily intake (%)		Contribution to safety dose (%)	
	Mean	Max	Mean	Max	Mean	Max
Fruit tea	0.3	3.46	2	24	6	69
Black tea	1.1	8.3	7	59	22	166
Coffee	0.26	0.36	1.82	2.52	5.2	7.2

## 3. Experimental Section

### 3.1. Chemicals and Columns

Hamilton DURACAL buffer solutions with a pH of 4.01 and 7.0 were purchased from Chromservis (Prague, Czech Republic). Hydrochloric acid, magnesium sulphate hexahydrate, glacial acetic acid, sodium chloride, kalium chloride, orthophosphoric acid 85%, anhydrous sodium acetate (all in p.a. purity), and methanol and acetonitrile (both in gradient grade for HPLC) were obtained from Merck (Prague, Czech Republic). Chloroform and sodium hydrogencarbonate (both p.a.) were purchased from Riedel-de Haën (Sigma-Aldrich spol. s r.o., Prague, Czech Republic). Phosphate buffer 10 mmol/L and OTA were obtained from Sigma (Sigma-Aldrich spol. s r.o., Prague, Czech Republic).

Phenylsilane strata phenyl SPE cartridges (55 µm, 70A) were manufactured by Phenomenex USA and delivered by Chromservis.cz (Prague, Czech Republic). Immunoaffinity columns Ochraprep for sample cleanup prior to the quantitative determination of OTA by HPLC were manufactured by R-Biopharm (Darmstadt, Germany) and delivered by Jemo Trading s r.o. (Bratislava, Slovak Republic). Whatman No. 4 paper filters from Merck (Prague, Czech Republic) and KA2-M (Papirna Pernštejn s r.o. (Pernštejn, Czech Republic)) were used for sample filtration. Sample extracts were evaporated under nitrogen 4.7 (SIAD spol. s r.o., Branany u Mostu, Czech Republic). All solutions were prepared in ultrapure water Milli Q Plus (Millipore, Billerica, MA, USA). 

### 3.2. OTA Solution

OTA stock solution was prepared by dissolving 1000 μg of OTA in 5 mL of methanol, quantitatively transferred into a 100 mL volumetric flask and filled to 100 mL with solvent. A standard solution (40 ng OTA/mL) was prepared from this stock solution. Stock and standard solutions were stored at −20 °C. The shelf-life of these solutions was one year. All calibration solutions were prepared on a daily basis from working (standard) OTA solution [69]. 

### 3.3. Sampling of Tea and Coffee

The study involved randomly and not probabilistically collected samples of black and fruit tea and coffee. Twelve samples (70 g to 125 g) of black tea, 12 samples (70 g) of fruit tea, and 12 samples (250 g) of ground roasted coffee were purchased from retailers in 12 locations in the Czech Republic all year round (spring, summer, autumn, and winter). Purchased samples were first homogenized and properly tested for their homogenity according to ISO 13528, 2005, Annex B, “Homogeneity and stability checks of samples” [63].

An additional 36 samples of ground roasted coffee (approximately 150 g of each sample) were obtained from the Institute of Public Health in Usti nad Labem, National Reference Laboratory for Biomarkers of Mycotoxins and Mycotoxins in Food, Hradec Kralove. These samples came from the hygienic surveillance of mycotoxins within the framework of reference and expertise activities of the Czech Republic.

### 3.4. Preparation of Tea and Coffee Beverages

A black tea drink was prepared (six times) from a naturally contaminated tea bag containing a high OTA concentration (250 ng OTA/g of tea). One black tea bag (ingredients: black tea and lemon pericarp) of 1.75 g was brewed in 250 mL of boiling water for 3 min, in accordance with the producer’s recommendations, and then cooled to approximately 30 °C. 

A fruit tea drink was prepared (six times) from a fruit tea bag, naturally contaminated with a high OTA concentration of 104 ng OTA/g of tea. One fruit tea bag (ingredients: hibiscus, apple, rose hips, cinnamon, elderberries, liquorice) of 1.75 g was left to infuse in 250 mL of boiling water for 7 min, in accordance with the producer’s recommendations, and cooled to approximately 30 °C. 

Seven types of coffee drinks were made (six times each) from naturally contaminated coffee (0.92 ng OTA/g): 

(i) *false Turkish coffee* (7 g of ground roasted coffee were suspended in 100 mL of boiling water for approximately 10 min); 

(ii) *Turkish coffee* (7 g of ground roasted coffee powder was suspended in 100 mL of water and boiled very slowly in a pot (“cezve”), cooled, and re-boiled. This process was repeated three times (time approximately 10 min);

(iii-vii) coffee drinks were prepared using the automatic coffee machine AEG, EA 100, Warstein-Belecke, Germany. The preparations of these drinks are based on pressure, inducing percolation of a limited amount of hot water through the ground coffee cake, where the energy of the water pressure was spent within the cake itself [70]. To produce coffee drinks with a cream layer on the top, specified volumes of hot water at a temperature below boiling temperature (96 °C) under high pressure (15 bar, 3.6 mL/s) were applied for 8.1–18 s. Hot drink preparation was carried out in accordance with the recommendation of the Italian National Institute of Coffee [71];

(iii) *Lungo* (80 mL of water on 7 g of ground roasted coffee); 

(iv) *Americano* (60 mL of water on 7 g of ground roasted coffee and addition of 40 mL of water to the drink); 

(v) *Espresso* (30 mL of water on 7 g of ground roasted coffee); 

(vi) *Doppio* (50 mL of water on 14 g of ground roasted coffee);

(vii) *Ristretto* (20 mL of water on 7 g of ground roasted coffee); 

The drinks obtained from black and fruit teas or coffee were used as extracts for OTA analysis. 

### 3.5. Determination of pH of Infusion

The determination of the pH was carried out by measuring the potential difference between electrodes immersed in standard and test solutions. The standard solutions were assigned a definite pH value by convention. In the measurement of pH, glass electrodes were applied as they showed an immediate response to rapid changes of hydrogen ion concentrations even in poorly buffered solutions.

For the determination of the pH, the pH meter WTW (model 330 with combined glass electrode; WTW, Weilheim, Germany) was used. The pH measurements were carried out in triplicate.

### 3.6. OTA Extraction and Sample Cleanup

Purification (cleanup) was done on an imunoaffinity column according to the protocol of Ochraprep^®^ (R-Biopharm AG-IAC, Darmstadt, Germany) and (i) the method of Zimmerli and Dick (1995) [72]; and (ii) the official method recommended in the Czech Republic by the norm No. 560063 (CSN EN 14132) on the basis of the method of collaborative study [73], detailed below: 

(i) Ten milliliters of NaCl/H_3_PO_4_ buffer solution adjusted to pH 1.6 were added to 5 g of a homogenized ground roasted coffee sample, the solution was shaken and the mixture was extracted four times with 5 mL of chloroform. The chloroform phases were collected and evaporated. Then the residues were dissolved in 5 mL of chloroform, and then twice re-extracted by 5 mL of 1% sodium hydrogenocarbonate buffer. These aqueous phases were collected into a container containing 0.5 mL of concentrated formic acid (98%–100%) and 1 mL of chloroform. This phase was re-extracted four times with 1 mL of chloroform. The chloroform portions were collected and evaporated under a stream of nitrogen and the residue was gradually dissolved in 20 mL of PBS (Phosphate buffered saline solution, pH 7.4) −15% methanol (*v*/*v*). Pooled 20 mL of PBS-15% methanol (*v*/*v*) were loaded onto immunoaffinity OCHRAPREP columns (speed loading approximately 1 mL/min). The columns were washed with 20 mL of distilled water and dried for 30 s under nitrogen stream. Then OTA was slowly eluted with 4 mL of methanol, which was evaporated to dryness under nitrogen stream. The residue was dissolved in 500 µL of methanol [72]. 

(ii) Ten grams of homogenized black or fruit tea or ground roasted coffee, or a filtered solution of the drink, were purified on a phenyl silane column. OTA was recovered by the addition of 10 mL methanol/water (7/93, *v*/*v*), to which 30 mL of phosphate buffer solution (PBS) were added [73]. This mixture was passed through IAC—the immunoaffinity OCHRAPREP columns (Darmstadt, Germany) —with a speed of loading around 1 mL/min. The column was washed with 20 mL PBS. OTA was recovered by slow elution of 4.0 mL of methanol. The solution was evaporated to dryness under nitrogen stream and dissolved in 500 μL of methanol [73].

The validated and accredited (CSN EN ISO/IEC 17025) method of reversed phase high-performance liquid chromatography (HPLC) with fluorescence detection was applied for the OTA detection and the quantification of OTA in black and fruit tea, coffee samples, and drinks. Validation of the method was performed according to the protocol approved by the AOAC and to the principles of the ICH Guideline for high-performance liquid chromatography. This method was accredited by the Czech Accreditation Institute (CSN EN ISO/IEC 17025). In 2012, the laboratory where the determinations took place successfully participated in the FAPAS (Proficiency testing) No. 17108 (ochratoxin A in the coffee), assigned value of 6.14 (ng/g), result of 5.06 (ng/g), recovery of 83 (%), *z*-score: −0.8, laboratory No.18. 

A linear calibration curve was built by measuring the OTA peak area of six standard methanolic solutions (concentrations of 0.125, 0.250, 0.500, 1.000, 2.000, and 4.000 ng OTA/mL and blank sample (methanol)). Each point of the calibration curve was measured in triplicate. The OTA concentrations were quantified using the calibration curve. The recoveries of HPLC method ranged from 75% to 85% and the standard deviation of repeatability from 1.3% to 3.7% depending on the type of matrix. The limit of dection (LOD) was 0.1 ng/g and the limit of quantification (LOQ) was 0.35 ng/g.

### 3.7. Conditions of HPLC Analysis for OTA Determination

OTA was determined by high-performance liquid chromatography with fluorescence detection (HPLC-FLD). The liquid chromatography equipment consisted of vacuum degasser SCM 400, gradient pump P 2000, autosampler AS 3000 (all from Spectra System, San Jose, CA, USA), fluorescence detector 920 FP (Jasco, Tokyo, Japan), and Solvent saver 2907 (Jour Research, Onsala, Sweden). The separation was performed on the C18 analytical column (length 300.0 mm–4.6 mm /I.D.; internal diameter) filled with Spherisorb ODS 2 with particle size of 10 µm (Bischoff Chromatography, Leonberg, Germany) coupled with guard column (3.0–4.0 mm) filled with C18 with particle size of 5 µm (Phenomenex, Torrance, CA, USA). The calculations and evaluations of the analyses were processed on the computer with software CSW 32 (Data Apex, Prague, Czech Republic). The injection volume of the samples was 50 µL. For OTA determination, the mobile phase was methanol/acetonitrile/ 0.005 mol/L sodium acetate/acetic acid (300/300/400/14, *v*/*v*/*v*/*v*) with a flow rate of 1.5 mL/min. Fluorescence detection was performed at an excitation wavelength of 333 nm and an emission wavelength of 465 nm. The OTA concentrations were quantified using the calibration curve method. The retention time of OTA was between 6.9 and 7.1 min [74]. 

The confirmation of the presence of OTA in samples of tea and coffee beverages took place by analyzing the formation of methyl ester with borontrifluoride (BF_3_): 1 mL of the purified extract (where OTA was detected by the HPLC analysis) or 1 mL of the standard solution (4 ng/mL) were dried on EVATERM and redissolved in 1 mL of BF_3_. Samples were incubated for 15 min at 60 °C. The samples were analyzed under the same HPLC chromatographic conditions described in point 2.7. The retention time of methylester OTA was shifted from 14.7 to 14.9 min instead of 6.9–7.1 min for OTA [74].

### 3.8. OTA Intake Evaluation in Cups of Tea and Coffee

To evaluate the intake, the exact amount in a cup of tea or coffee (Qc) should be calculated taking into account the initial quantity of tea and coffee (“a” in ng/g) used for the infusion and the percentage of transfer (“*t*”). For (black and fruit) teas the amount used to prepare the infusion was 1.75 g. For coffee the amount used was 7 g (except for Doppio, where the amount was 14 g).

Qc_tea_ = *a* (ng/g) × 1.75 × *t* for tea (1)

Qc_coffee_ = *a* (ng/g) × 7 × *t* for coffee (2)

where *a* is the initial concentration of the tea or coffee expressed in ng/g and *t* the percentage of transfer. This value will directly serve to calculate the intake for an adult of 60 kg drinking the entire cup.

## 4. Conclusions

For the first time the occurence of OTA in black and fruit teas and the natural OTA transfer from black tea and fruit tea into infusions were analyzed. This small survey concerning OTA content in black and fruit tea pinpoints this supply as a non-negliglible contributor to OTA intake. Even though high contamination amount in black tea (250 ng/g) is not the rule, it was demonstrated that a significant amount of OTA can be transferred in black tea infusions (about 35%, whatever the initial concentration in the raw material). The OTA transfer in fruit tea infusions was much lower (4%) due to the presence of organic acid but in some cases was not negliglible, as with the most contaminated sample (104 ng/g) In addition, it should be kept in mind that some consumers drink more then one cup per day.

Let us suppose that a person with an average body weight of 60 kg consumes 3–5 cups of hot drinks; considering the overall mean and maximum values observed in this study, coffee consumption contributed to 1.82%–2.5% of the TDI, fruit tea to 2%–24%, and black tea to 7%–59%.

The high variability between beverages is influenced by the quality of the raw material (initial amount of OTA in raw tea or coffee), but also by the pH of the drinks, the temperature of the water, and the time of contact.

Only a limited number of tea samples were analyzed. High batch variation was observed, demonstrating the need for an accurate quality control of the raw materials used for the infusions. In the future, it will be crucial to do a larger survey to be able to make an accurate risk assessment. 

In addition, focus should be put on other OTA derivatives, as analysis of chromatograms of infusion notably pointed to the presence of OTB (dechlorinated OTA) and other OTA derivatives such as 14[R]-OTA and decarboxylated OTA (data not shown). It should be remembered, however, that the disappearance (conversion) of OTA does not necessarily mean an absence or decrease in toxicity risk as the decomposition products may be as dangerous as the parent OTA molecule itself.

Thus, for more accurate toxicological assessment, determination of all ochratoxin derivatives in tea and coffee drinks should be conducted. 

In order to minimize the health risks resulting from the consumption of tea or coffee infusions contaminated by toxigenic fungi and mycotoxins, it is imperative to monitor the presence of these contaminants. 

## References

[1] Esquivel P., Jiménez V.M. (2012). Functional properties of coffee and coffee by-products. Food Res. Int..

[2] Bisht S., Sisodia S.S. (2010). Coffea arabica: A wonder gift to medical science. J. Nat. Pharmaceut..

[3] Farah A. (2009). Coffee as a speciality and functional beverage. Paquin, p. Speciality and Functional Beverages.

[4] Vignoli J.A., Bassoli D.G., Benassi M.T. (2011). Antioxidant activity, polyphenols, caffeine and melanoidins in soluble coffee: The influence of processing conditions and raw material. Food Chem..

[5] Pan M.H., Lai Ch.S., Wang H., Loc Ch.Y., Hod Ch.T., Li S. (2013). Black tea in chemo-prevention of cancer and other human diseases. Food Sci. Hum. Wellness.

[6] Farah A., Donagelo C.M. (2006). Phenolic compounds in coffee. Brazil J. Plant Physiol..

[7] Meletis C.D. (2006). Coffee—Functional food and medicinal herb. Alter. Compl. Ther..

[8] Pfohl-Leszkowicz A., Manderville R.A. (2007). Ochratoxin A: An overview on toxicity and carcinogenicity in animals and humans. Mol. Nutr. Food Res..

[9] Pfohl-Leszkowicz A., Manderville R.A. (2012). An update on direct genotoxicity as a molecular mechanism of ochratoxin A carcinogenicity. Chem. Res. Toxicol..

[10] Malir F., Ostry V., Novotna E. (2013). Toxicity of the mycotoxin ochratoxin A in the light of recent data. Toxin Rev..

[11] Jørgensen K. (1998). Survey of pork, poultry, coffee, beer and pulses for ochratoxin A. Food Addit. Contam..

[12] Santos L., Marin S., Sanchis V., Ramos A.J. (2009). Screening of mycotoxin multicontamination in medicinal and aromatic herbs sampled in Spain. J. Sci. Food Agric..

[13] Van der Merwe K.J., Steyn P.S., Fourie L., Scott De B., Theron L.L. (1965). Ochratoxin A, a toxic metabolite produced by *Aspergillus ochraceus* Wilh. Nature.

[14] Abarca M.L., Bragulat M.R., Castella G., Cabañes F.J. (1994). Ochratoxin A production by strains of *Aspergillus niger* var *niger*. Appl. Environ. Microbiol..

[15] Mantle P.G., Chow A.M. (2000). Ochratoxin formation in *Aspergillus ochraceus* with particular reference to spoilage of coffee. Int. J. Food Microbiol..

[16] Samson R.A., Frisvad J.C. (2004). *Penicillium* subgenus *Penicillium*: New taxonomic schemes and mycotoxins and other extrolites. Stud. Mycol..

[17] Tozlovanu M., Pfohl-Leszkowicz A. (2010). Ochratoxin A in roasted coffee purchased in french super market. Transfer in coffee beverage: Comparison of several methods. Toxins.

[18] Skarkova J., Ostry V., Malir F., Roubal T. (2013). The determination of ultra-trace amounts of ochratoxin A in foodstuffs by HPLC method. Anal. Lett..

[19] Ostry V., Malir F., Ruprich J. (2013). Producers and important dietary sources of ochratoxin A and citrinin. Toxins.

[20] Gil-Serna J., Vázquez C., Sandino F.G., Valle A.M., González-Jaén M.T., Patiño B. (2014). Evaluation of growth and ochratoxin A production by *Aspergillus stenii* and *Aspergillus westerdijkiae* in green-coffee based medium under different environmental conditions. Food Res. Int..

[21] Taniwaki M.M.H., Teixeira A.A., Teixeira A.A.R., Copetti M.V., Iamanaka B.T. (2014). Ochratoxigenic fungi and ochratoxin A in defective coffee beans. Food Res. Int..

[22] Pitt J.I., DeVries J.W., Truckseess M.W., Jackson L.S. (2002). Biology and ecology of toxigenic *Penicillium* species. Mycotoxins and Food Safety.

[23] Petzinger E., Ziegler K. (2000). Ochratoxin A from a toxicological perspective. J. Vet. Pharmacol. Ther..

[24] Abdel-Hafez A.I.I., El-Maghraby O.M.O. (1992). Fungal flora and aflatoxin associated with cocoa, broasted coffee and tea powders in Egypt. Crypt. Mycol..

[25] Aziz N.H., Youssef Y.A., El-Fouly M.Z., Moussa L.A. (1998). Contamination of some common medicinal plant samples and species by fungi and their mycotoxins. Bot. Bull. Acad. Sin..

[26] Halt M. (1998). Moulds and mycotoxins in herb tea and medicinal plants. Eur J. Epidemiol..

[27] Elshafie A.E., Al-Lawatia T., Al-Bahry S. (1999). Fungi associated with black tea and tea quality in the Sultanate of Oman. Mycopathologia.

[28] Ostry V., Ruprich J., Skarkova J., Prochazkova I., Kubatova A. (2001). Occurrence of the toxigenic fungi (Producers of aflatoxins and ochratoxin A) in foodstuffs in the Czech Republic 1999–2000. Mycotox. Res..

[29] Ostry V., Ruprich J., Skarkova J., Prochazkova I., Kubatova A. (2002). MYCOMON—Monitoring project of toxigenic fungi in food in years 1999–2000. Mycotox. Res..

[30] Rezacova V., Kubatova A. (2005). Saprobic microfungi in tea based on *Camellia sinensis* and on other dried herbs. Czech Mycol..

[31] Abd El-Atya A.M., Choia J.-H., Musfiqur R.M.D., Kim S.-W., Tosun A., Shima J.-H. (2014). Residues and contaminants in tea and tea infusions. Food Addit. Contam..

[32] Tournas V.H., Katsoudas E.J. (2008). Microbiological quality of various medicinal herbal teas and coffee substitutes. Microbiol. Insights.

[33] Storari M., Dennert F.G., Bigler L., Gessler C., Broggini G.A.L. (2012). Isolation of mycotoxins producing black *Aspergilli* in herbal teas available on the Swiss market. Food Control.

[34] Haas D., Pfeifer B., Reiterich Ch., Partenheimer R., Reck B., Buzina W. (2013). Identification and quantification of fungi and mycotoxins from Pu-erh tea. Int. J. Food Microbiol..

[35] Levi C.P., Trenk H.L., Mohr H.K. (1974). Study of the occurrence of Ochratoxin A in green coffee beans. J. AOAC.

[36] Micco C., Grossi M., Miraglia M., Brera C. (1989). A study of the contamination by ochratoxin A in green coffee and roasted coffee beans. Food Addit. Contam..

[37] Van der Stegen G., Jorissen U., Pittet A., Saccon M., Steines W., Vincenzi M., Winkler M., Zapp J., Schlatter C. (1997). Screening of European coffee final products for occurrence of ochratoxin A. Food Addit. Contam..

[38] Nakajima M., Tsubouchi H., Miyabe M., Ueno Y. (1997). Survey of aflatoxin B_1_ and ochratoxin A in commercial green coffee beans by high-performance liquid chromatography linked with immunoaffinity chromatography. Food Agric. Immunol..

[39] Trucksess M., Giler J., Young K., White K.D., Page S.W. (1999). Determination and survey of ochratoxin A in wheat, barley and coffee 1997. J. AOAC Int..

[40] Romani S., Sacchetti G., Chaves C.C., Pinnavaia G.G., Dalla M. (2000). Screening on the occurrence of ochratoxin A in green coffee beans of different origins and types. J. Agric. Food Chem..

[41] Miraglia M., Brera C. (2002). Assessment of dietary intake of ochratoxin A by the population of EU Member States. Report on Tasks for Scientific Cooperation.

[42] Taniwaki M.H., Pitt J.I., Teixeira A.A., Iamanaka B.T. (2003). The source of ochratoxin A in Brazilian coffee and its formation in relation to processing methods. Int. J. Food Microbiol..

[43] Martins M.L., Martins H.M., Gimeno A. (2003). Incidence of microflora and of ochratoxin A in green coffee beans (*Coffea arabica*). Food Addit. Contam..

[44] Drunday V., Pacin A. (2013). Occurrence of Ochratoxin A in coffee beans, ground roasted coffee and soluble coffee and method validation. Food Control.

[45] Patel S., Hazel C.M., Winterton A.G.M., Gleadle A.E. (1997). Survey of ochratoxin A in UK retail coffees. Food Addit. Contam..

[46] Burdaspal P.A., Legarda T.M. (1998). Ochratoxin A in roasted and soluble coffee marketed in Spain. Alimentaria.

[47] Coronel M.B., Marin S., Cano G., Ramos A.J., Sanchis V. (2011). Ochratoxin A in Spanish retail ground roasted coffee: Occurrence and assessment of the exposure in Catalonia. Food Control.

[48] Galarce-Bustos O., Alvarado M., Vega M., Aranda M. (2014). Occurrence of ochratoxin A in roasted and instant coffees in Chilean market. Food Control.

[49] Paterson R.R.M., Lima N., Taniwaki M.H. (2014). Coffee, mycotoxins and climate change. Food Res. Int..

[50] Iamanaka B.T., Teixeira A.A., Teixeira A.R.R., Copetti M.V., Bragagnolo N., Taniwaki M.H. (2014). Reprint of “The mycobiota of coffee beans and its influence on the coffee beverage”. Food Res. Int..

[51] Castellanos-Onorio O., Gonzalez-Rios O., Guyot B., Fontana T.A., Guiraud J.P., Schorr-Galindo S., Durand N., Suárez-Quiroz M. (2011). Effect of two different roasting techniques on the Ochratoxin A (OTA) reduction in coffee beans (Coffea arabica). Food Control.

[52] Rezende E.F., Borges J.G., Cirillo M.A., Prado G., Paiva L.C., Batista L.R. (2013). Ochratoxigenic fungi associated with green coffee beans (*Coffea arabica* L.) in conventional and organic cultivation in Brazil. Brazil. J. Microbiol..

[53] Studer-Rohr I., Dietrich D.R., Schlatter J., Schlatter C. (1995). The occurrence of ochratoxin A in coffee. Food Chem. Toxicol..

[54] Perez de Obamos A., Gonzalez-Penas E., Lopez de Cerain A. (2005). Influence of roasting and brew preparation on the ochratoxin A content in coffee infusion. Food Addit. Contam..

[55] Santini A., Ferracane R., Mikusova P., Eged S., Srobarova A., Meca G., Mańes J., Ritieni A. (2011). Influence of different coffee drink preparations on ochratoxin A content and evaluation of the antioxidant activity and caffeine variations. Food Control.

[56] Krug H.P. (1940). Cafés Duros, III. Relação entre a porcentagem de microrganismos e qualidade do café. Revista do Instituto do Café.

[57] Krug H.P. (1941). Cafés Duros, IV. Relação entre zonas, qualidade de café e porcentagem de microrganismos. Revista do Instituto do Café.

[58] Carvalho V.D., Chalfoun S.M. (1985). Aspectos qualitativos do café. Informe Agropecuário.

[59] Pardo E., Marin S., Ramos A.J., Sanchis V. (2004). Occurrence of ochratoxigenic fungi and ochratoxin A in green coffee from different origins. Food Sci Technol. Int..

[60] Frisvad J.C., Thrane U., Samson R.A., Pitt J.I. (2006). Important mycotoxins and the fungi which produce them. Adv. Food Mycol..

[61] Schmidt-Heydt M., Bode H., Raupp F., Geisen R. (2010). Influence of light on ochratoxin biosynthesis by *Penicillium*. Mycotox. Res..

[62] La Pera L., Avellone G., Lo Turco V., Di Bella G., Agozzino P., Dugo G. (2008). Influence of roasting and different brewing processes on the Ochratoxin A content in coffee determined by high-performance liquid chromatogramy fluorescence detection (HPLC-FLD). Food Addit. Contam..

[63] Statistical methods for use in proficiency testing by interlaboratory comparisons. http://www.iso.org/iso/catalogue_detail.htm?csnumber=56125.

[64] Belitz H.D., Grosch W., Schieberle P. (2011). Food Chem.

[65] Perrone D., Farah A., Donangelo C.M. (2012). Influence of coffee roasting on the incorporation of phenolic compounds into melanoidins and their relationship with antioxidant activity of the brew. J. Agric. Food Chem..

[66] Mills Ch.E., Oruna-Concha M.J., Mottram D.S., Gibson G.R., Spencer J.P.E. (2013). The effect of processing on chlorogenic acid content of commercially available coffee. Food Chem..

[67] Ostry V., Skarkova J., Malir F. (2014). Ochratoxin A—health risk assessment for selected population groups in the Czech Republic.

[68] Kuiper-Goodman T., Hilts C., Billiard S.M., Kiparissis Y., Richard I.D.K., Hayward S. (2010). Health risk assessment of ochratoxin A for all age-sex strata in a market economy. Food Addit. Contam..

[69] Dohnal V., Dvorak V., Malir F., Ostry V., Roubal T. (2013). A comparison of ELISA and HPLC methods for determination of Ochratoxin A in human blood serum in the Czech Republic. Food Chem. Toxicol..

[70] Caporaso N., Genovese A., Canela M.D., Civitella A., Sacchi R. (2014). Neapolitan coffee brew chemical analysis in comparison to espresso, moka and American brews. Food Res. Int..

[71] Odello L., Odello C. (2009). Espresso Italiano Tasting.

[72] Zimmerli B., Dick R. (1995). Determination of ochratoxin A at the ppt level in human blood, serum, milk and some foodstuffs by high, performance liquid chromatography with enhanced fluorescence detection and immunoaffinity column cleanup: Methodology and Swiss data. J. Chromatogr. B.

[73] Entwisle A.C., Williams A.C., Mann P.J., Russell J., Slack P.T., Gilbert J. (2001). Combined phenyl silane and immunoaffinity column clean-up with liquid chromatography for determination of ochratoxin A in roasted coffee: Collaborative study. J. AOAC Int..

[74] Creppy E.E., Castegnaro M., Grosse Y., Meriaux J., Manier C., Moncharmont P., Waller C., Creppy E.E., Castegnaro M., Dirheimer G. (1993). Etude De L’ochratoxicose Humaine Dans Trois Regions de France. Alsace, Aquitaine et Region Rhone-Alpes. Human Ochratoxicosis and Its Pathologies.

